# Plant probiotic bacteria: solutions to feed the world

**DOI:** 10.3934/microbiol.2017.4.747

**Published:** 2017-08-24

**Authors:** Esther Menendez, Paula Garcia-Fraile

**Affiliations:** 1 Instituto de Ciências Agrárias e Ambientais Mediterrânicas (ICAAM), Universidade de Évora, Évora, Portugal; 2 Laboratory of Fungal Genetics and Metabolism, Institute of Microbiology, Academy of Sciences of the Czech Republic, Prague, Czech Republic; 3 Department of Microbiology and Genetics, University of Salamanca, Salamanca, Spain

Plant probiotic bacteria represent a heterogeneous group of prokaryotes, mostly natural-occurring soil bacteria, which have beneficial traits directly related to enhance plant development. Specifically, this kind of beneficial bacteria, acting as plant symbionts or plant endophytes, enhances plant growth, crop yields and their products quality (grains, fruits and/or others).

Over the last decades, a plethora of studies have focused on the isolation of potential plant probiotic bacteria from soil, rhizosphere or plant tissues. Moreover, their applications and efficiency as single inoculants or as a part of bacterial consortia have been evaluated in several kind of crops, such as cereals, legumes, vegetables and other kind of superior plants, such as trees, ornamentals and aromatic plants.

This special issue pretends to cover established and updated studies about plant probiotic bacteria and their benefits for agriculture and forestry, which will have a direct and beneficial impact on human and animal health and also, will minimize the use of chemical fertilizers, contributing to a more sustainable agriculture and forestry.

This Special Issue contains 15 publications, 10 reviews and 5 original research articles, which cover most of the current aspects related to the benefits of plant probiotic bacteria in the global agriculture, combining recent basic and applied knowledge about this topic.

The content of this Special Issue can be divided in six different parts: (i) Applications of plant probiotic bacteria in agriculture and forestry, with 3 contributions (Ishaq et al., Porto de Souza Vandenberghe et al., Cruz-Gonzalez et al.); (ii) Taxonomy and systematics of Plant Probiotic Bacteria in the “genomics” era, with 1 contribution (Carro and Nouioui); (iii) Effects on yield and quality of crops and fruits, with 4 contributions (Di Benedetto et al., Jimenez-Gomez et al., Diez-Mendez and Rivas, Silva et al.,); (iv) Effects on biocontrol and bioremediation, with 3 contributions (Ortiz et al., Valverde et al. and Veliz et al.); (v) plant probiotic bacteria interactions with their hosts and environment, with 3 contributions (Hidalgo et al., Da-Silva et al. and Muñoz-Azcarate et al.) and (vi) Technologies and current marketing of PPB-based products, with 1 contribution (Menendez and García-Fraile).

The editors acknowledge all the authors for their valuable and straightforward contributions to this Special Issue.

